# PDLSCs Regulate Angiogenesis of Periodontal Ligaments via VEGF Transferred by Exosomes in Periodontitis

**DOI:** 10.7150/ijms.40918

**Published:** 2020-02-10

**Authors:** Zhang Zhang, Yi Shuai, Feng Zhou, Jikai Yin, Jiachen Hu, Songlin Guo, Yan Wang, Wenjia Liu

**Affiliations:** 1 Department of General Surgery, Tang Du Hospital, Fourth Military Medical University, Xi'an, Shaanxi 710032, People's Republic of China;; 2 Department of Stomatology, Jinling Hospital, Medical School of Nanjing University, Nanjing, Jiangsu 210002, People's Republic of China;; 3 State Key Laboratory of Military Stomatology & National Clinical Research Center for Oral Diseases & Shaanxi International Joint Research Center for Oral Diseases, Center for Tissue Engineering, School of Stomatology, Fourth Military Medical University, Xi'an, Shaanxi 710032, People's Republic of China;; 4 Department of Stomatology, General Hospital of Eastern Theater Command, PLA, Nanjing, Jiangsu 210002, People's Republic of China;; 5 Xi'an Institute of Tissue Engineering and Regenerative Medicine, Xi'an, Shaanxi 710032, People's Republic of China;; 6 Department of Clinical Laboratory, The First Affiliated Hospital of Xi'an Medical University, Xi'an, Shaanxi 710032, People's Republic of China.

**Keywords:** periodontitis, angiogenesis, PDLSCs, exosomes, miR-17-5p, VEGF

## Abstract

Abnormal angiogenesis is one of the significant features in periodontitis leading to progressive inflammation, but angiogenic changes of periodontal ligaments under inflammatory condition were rarely reported. Periodontal ligament stem cells (PDLSCs) were a kind of dental stem cells associated with vascularization. Here we investigated the alteration of angiogenesis of periodontal ligament in periodontitis, and revealed an exosome-mediated pathway to support the effect of PDLSCs on angiogenic improvement. Vascular specific marker CD31 and VEGFA were found to be highly expressed in periodontal ligaments of periodontitis. The VEGFA expression was up-regulated in inflamed PDLSCs compared to control, meanwhile the tube formation of HUVECs was improved when co-cultured with inflamed PDLSCs. Exosomes secretion of PDSLCs was augmented by inflammation, and promoted angiogenesis of HUVECs, whereas blocking secretion of exosomes led to degenerated angiogenesis of HUVECs. Exosome-trasferred VEGFA was proven to be the crucial communicator between PDLSCs and HUVECs. Inflammation inhibited miR-17-5p expression of PDLSCs and relieved its target VEGFA. However, overexpression of miR-17-5p blocked the pro-angiogenic ability of inflamed PDLSCs. In conclusion, the findings indicated that vascularization of periodontal ligaments was enhanced, and inflammatory micro-environment of periodontitis facilitated pro-angiogenesis of PDLSCs through regulating exosome-mediated transfer of VEGFA, which was targeted by miR-17-5p.

## Introduction

Periodontitis is identified as a chronic bacterial infectious disease, which invades the tooth-supporting tissues, such as gingiva, periodontal ligaments and alveolar bone 1. The growth and remodeling of the skeletal system are generally coupled with angiogenesis, including alveolar bone 2. Anomalous vascularization is one of the most common characteristics in periodontitis leading to progressive inflammation, which results from disrupted angiogenesis in the periodontal tissues 3. Recently, large numbers of studies on periodontitis have been concentrating on angiogenesis in the inflammatory tissues 4. Increased blood vessels were observed in gingiva of the periodontitis as a result of enhanced inflammatory infiltration 5, 6. Periodontal ligament locates in the gap between tooth root and alveolar bone to fasten the tooth, which is often invaded and destructed by inflammation when chronic periodontitis was extending 7. Therefore it might be very important to determine the alteration of periodontal ligament for uncovering pathogenesis of periodontitis. However, changes of vascularization in periodontal ligament were rarely reported in the condition of periodontitis, and detailed mechanisms remain to be revealed.

Mesenchymal stem cells (MSCs) have been widely reported to be associated with angiogenesis, including dental stem cells 4, 8, 9. Periodontal ligament stem cells (PDLSCs) are a class of odontogenic MSCs residing in periodontal ligament with functions of self-renewal, multi-directional differentiation potential and cellular regulation 10. Recent study reported that PDLSCs had an ability of angiogenic promotion 4, 9, whereas the underlying mechanism of PDLSCs on angiogenesis under inflammatory micro-environment needed to be uncovered.

Paracrine is an important mode of intercellular communication, which is achieved by secreting soluble factors, protein complex and packaged mediators 11, 12. Exosomes, a type of packaged mediators probably containing functional proteins, functionally transfers to other cells through such an emerging novel paracrine pathway 12, 13. Numerous evidences showed that MSCs, as well as dental stem cells, could regulate function of endothelial cells via exosome-mediated signaling 14, 15. Various biological factors were involved in angiogenic regulation 16. However, the vascular endothelial growth factor (VEGF) was commonly regarded as the most potent agent participating in modulation of vascular endothelium 17. Furthermore, VEGF was reported to be a major stimulant in the process of coupling between osteogenesis and angiogenesis 18, 19.

Numerous micro-RNAs (miRNAs) were investigated to play significant roles in dental tissues 20. MiR-17, an inhibitor of VEGF, is a crucial microRNA associated with bone remodeling and angiogenesis 21-24. Our previous study demonstrated that miR-17 regulated periodontal tissue regeneration via PDLSCs-mediated pathway in chronic periodontitis 21, 22, 24. Therefore, current work was assumed to explore whether PDLSCs can regulate angiogenesis via exosome-mediated VEGF communication under inflammatory environment of periodontitis.

In current study, enhanced vascularization was found in periodontal ligament, as well as gingiva, derived from chronic periodontitis. PDLSCs cultured in inflammatory condition expressed more VEGFA and promoted tube formation of human umbilical vein endothelial cells (HUVECs). Additionally, blocking of exosomes secretion suppressed the tube formation, and inflammation inhibited expression of miR-17-5p while improved the VEGFA expression in exosomes. All the observations suggested that PDLSCs, under inflammatory condition of periodontitis, regulated angiogenesis through exosome-mediated transportation of VEGFA targeted by miR-17-5p, which uncovered a novel mechanism of aberrant vascularization in periodontal ligaments of periodontitis and proposed a therapeutic target for periodontitis.

## Materials and Methods

### Animal model of periodontitis

Twenty adult female Sprague-Dawley rats (200.4±25.3g) were purchased from the Laboratory Animal Research Centre of the Fourth Military Medical University. Periodontitis was induced by injection of *Escherichia Coli* LPS (10 μL, 1 mg/mL) at the mediolateral of the left mandibular first molar, while the control group received 10 μL of saline. This administration was repeated every other day until 30 days. All the procedures were approved by Animal Care Committee of the Fourth Military Medical University, following the NIH Guide for the Care and Use of Laboratory Animals.

### Micro-CT analysis

The micro-CT system (L-sp, GE, USA) was applied to scan the mandible samples with a source voltage of 80 kV, current of 500 mA and isotropic resolution of 14.97 mm. Three-dimensional images of the periodontal defects were reconstructed by the supporting software of micro-CT.

### Immunofluorescent analysis

Frozen sections of periodontal tissues were prepared for immunofluorescent staining. The gingiva and periodontal ligament were tested by immunofluorescence against CD31 (ab119339) and VEGF (ab46154) (Abcam, USA) 30 days after induction of periodontitis. Sections were stained using primary antibodies at 4℃ overnight. The nuclei were then stained using DAPI at 37℃ for 30 min after incubation with secondary antibodies of Alexa Fluor 488 and Alexa Fluor 594.

### Patients and tissue samples

Impacted premolars of patients were collected at the School of Stomatology, Fourth Military Medical University, whose teeth were extracted because of orthodontic purposes or periodontitis. All the procedures of human samples were approved by the Institutional Review Board for Human Subjects Research of Fourth Military Medical University (IRB-REV-2016021). All the patients signed informed consent to this study.

### Cell isolation, culture and differentiation assay

Periodontal ligaments derived from normal and periodontitis donors were separated from the middle of root surfaces, then were cut into small pieces (about 1 mm^3^) and digested using collagenase type I (3 mg/mL), dispase (4 mg/mL) (Sigma Aldrich, USA) for 15 min. Single-cell suspensions (2×10^3^ cells) were seeded and cultured in α-MEM (Gibco, USA) with 10% FBS (Gibco), streptomycin (100 IU/ml; Gibco) and penicillin (100 μg/ml; Gibco) with an environment of 37℃ and humidified 5% CO_2_. PDLSCs were cultured in basic medium until reaching 80% confluence, then were induced by osteogenic condition medium for 28 days or adipogenic condition medium for 14 days. Alizarin Red and Oil O Red were used for staining of mineralized nodules or lipid droplets. The 2^nd^-5^th^ passages of PDLSCs were used for each experiment.

### Flow cytometry

The single-cell suspension of 5×10^5^ PDLSCs (P5) was incubated with antibodies for human Stro-1 (PE), CD146 (PE), CD105 (PE), CD29 (FITC), CD34 (PE) and CD45 (APC) (BD Bioscience, USA) at 4°C without light. The samples were detected by flow-cytometry using a Beckman Coulter Epics XL cytometer (Beckman Coulter, USA) to identify stem cell surface markers.

### Exosomes isolation

PDLSCs were cultured with vesicle-free medium and serum. The residual cells were removed from harvested conditioned medium by centrifuging for 10 min at 500 g. Next, the apoptotic bodies and debris were removed by centrifugation of the supernatant for 30 min at 16,000 g. A following ultracentrifugation for 70 min at 150,000 g (Beckman Optima XPN-100, USA) was conducted to collect exosomes. The exosomes were stored at 80℃ in PBS for further research.

### Morphological examination of exosomes

The isolated exosomes were suspended and dyed using Phosphotungstic Acid (PTA). The morphology of exsomes was observed and captured by transmission electron microscopy (Tecnai G2, USA).

### Tube formation assay

Matrigel (BD Biosciences, USA) and a 24-well transwell plate (Corning, NY) were used for tube formation assay. 2×10^4^ HUVECs per well were seeded in matrigel-coated 24-well plate, and indirectly co-cultured with 1×10^4^ PDLSCs or 50 μg/mL exosomes for 5 h at 37℃. After incubation, tube formation was determined under optical microscope (Leica, Germany). The network structures of tube formation were measured using Image J software.

### Transfection of miRNA mimics and inhibitor

Cells were used for transfection when reached to 65% confluence. PDLSCs were transfected with miR-17-5p mimics at 20 nM and inhibitor at 60nM (RiboBio, China) using Ribo FECT CP Transfection Kit (RiboBio, China) according to the instructions. The transfection medium was replaced after 8 h, and the cells were collected for protein analysis after 48 h.

### Luciferase Reporter Assay

Luciferase reporter assay was performed as reported previously 22. In brief, VEGFA oligonucleotide sequences were amplified using the primers (sense: 5′-ATCTTCAAGCCGTCCTGTG-3′, anti-sense: 5′-GAATGGGTTTGTCGTGTTT-3′) with SpeIand HindIII sites at their ends to insert pMIR-Report luciferase plasmid (Ambion, USA). The pMIR-control, pMIR-VEGFA plasmids were used as reporter constructs, and a Renilla luciferase reporter without miRNA binding sites was used as the loading control. All plasmids were co-transfected into PDLSCs with miR-17-5p mimics, inhibitor, or negative control using Lipofectamine 2000 (Invitrogen, USA). After 48 h, luciferase activities were determined using a dual-luciferase reporter assay kit (Promega, USA) following the protocols.

### Western blot analysis

Protein concentration measurement was conducted using a BCA kit (Beyotime Biotechnology, China). Equal protein amount from each sample was taken for western blot analysis. The proteins were transferred to PVDF membrane after separation. 5% non-fat milk powder solution was used to block non-specific bands. Antibody of VEGF (ab46154) and TSG101 (ab125011) (Abcam, USA), GAPDH (CW0100) and β-Tubulin (CW0098) (CWBio, China), CD63 (sc-5275) (Santa Cruz Biotechnology, USA) were used for western blot. The protein bands were developed using Amersham Imager 600 (GE Healthcare Life Sciences, USA).

### Statistical analysis

All the data were displayed using mean ± SEM and were analyzed by Student's t test or one-way ANOVA for comparison of two or multiple groups with SPSS software v11.0 (SPSS, USA). P<0.05 was considered as statistically significant.

## Results

### The vascularization of gingiva and periodontal ligaments were both up-regulated in periodontitis

**Figure 1:**
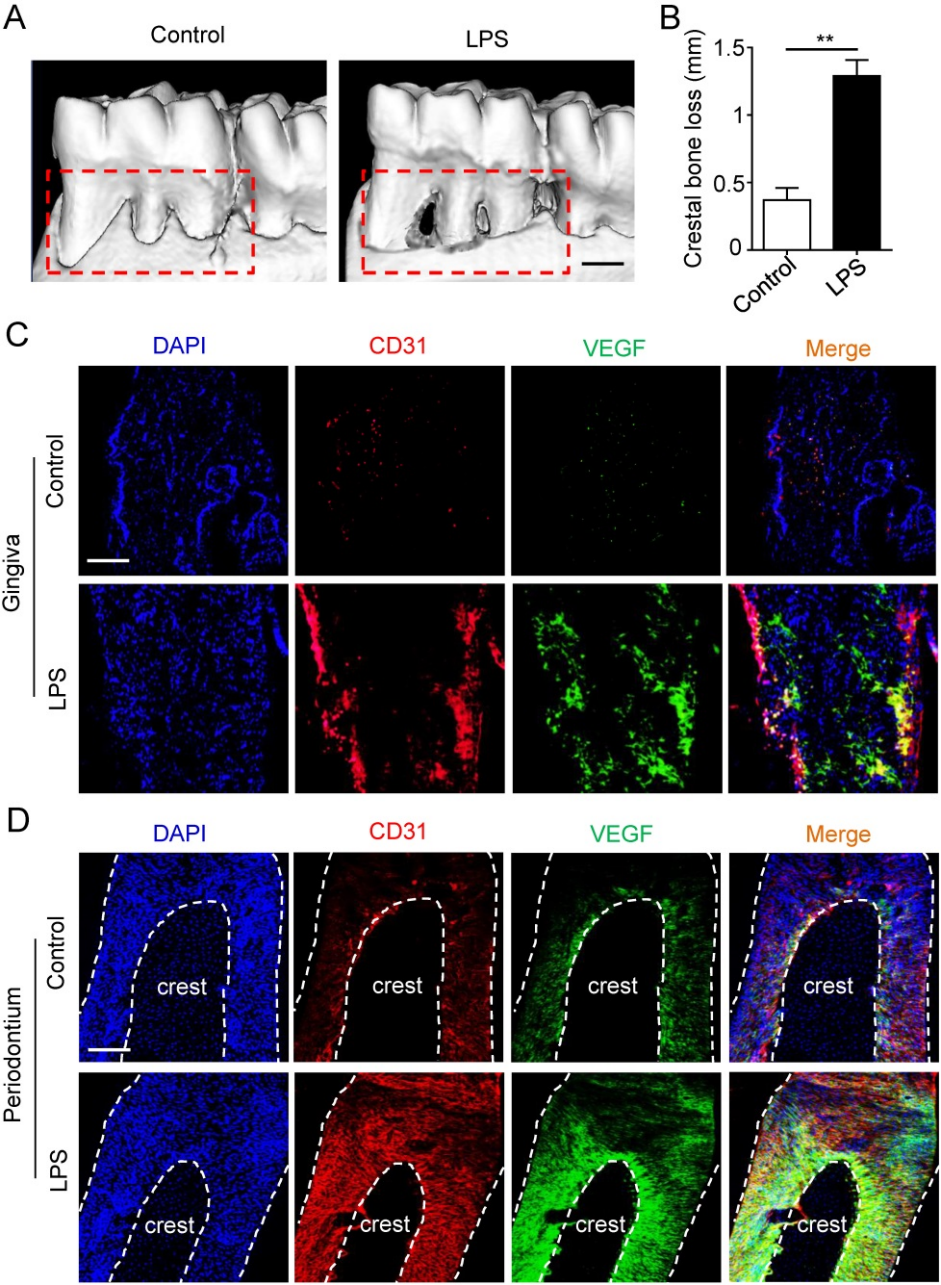
** The angiogenesis of gingiva and periodontal ligaments in periodontitis.** The alveolar bone (A) and crestal bone loss (B) were analyzed using micro-CT and quantitative analysis of the crestal bone loss (n = 6). Scale bar = 500 μm. Vascular markers CD31 and VEGF of gingiva (C) and periodontal ligaments (D) were stained using immunofluorescent analysis. Scale bar = 200 μm. **p<0.01. Unpaired two-tailed Student's t-test.

Micro-CT was applied to estimate the periodontitis model. Periodontal defects were observed in periodontitis group (Fig. 1A), meanwhile the crestal bone loss of periodontitis group was increased compared to control group (Fig. 1B), suggesting that periodontitis model was successfully established. Furthermore, to investigate the angiogenesis of gingiva and periodontal ligaments, tissues were stained by vascular marker CD31 and VEGFA. Compare with control group, the positive areas of CD31 and VEGFA were larger in gingiva of periodontitis group (Fig. 1C), as well as the overlapped area of the two markers. In addition, similar results were observed in periodontal ligaments (Fig. 1D), which indicated that periodontal ligaments shared similar vascular alteration that was up-regulated in periodontitis.

### The pro-angiogenic capacity of PDLSCs was improved under inflammatory micro-environment

**Figure 2:**
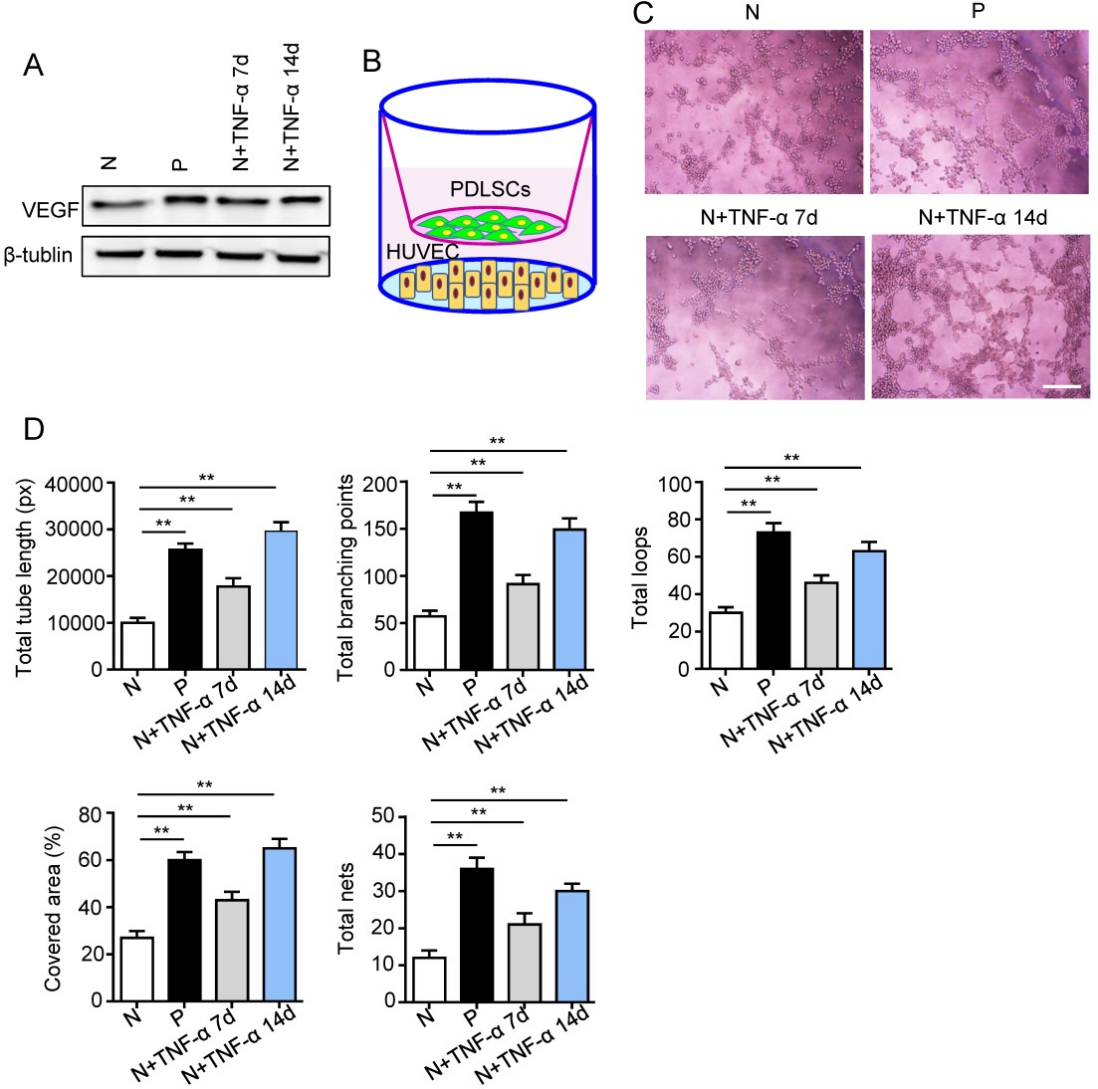
** The pro-angiogenic capacity of PDLSCs under inflammatory micro-environment.** (A) VEGF expression of PDLSCs was detected using western blot. (B) A transwell co-culture system was applied. Tube formation of HUVECs (C) was observed under microscope after co-culturing with PDLSCs, and total tube length, total branching points, total loops, covered area and total nets (D) were analyzed using Image J. Scale bar = 200 μm. **p<0.01. One-way analysis of variance (ANOVA).

Surface markers, osteogenesis and adipogenesis of PDLSCs were identified (Fig. S1). To determine the effect of inflammatory micro-environment on pro-angiogenic capacity of PDLSCs, the expression of VEGFA was examined using western blot. The VEGFA was up-regulated in PDLSCs derived from periodontitis and PDLSCs treated with inflammatory factors compared to control group (Fig. 2A). Moreover, a transwell co-culture system was employed to evaluate the effect of PDLSCs on HUVECs (Fig. 2B). The tube formation ability, including total tube length, total branching points, total loops, covered area and total nets, was enhanced in PDLSCs derived from periodontitis and PDLSCs treated with inflammatory factors compared to control group (Fig. 2C and D). Additionally, the VEGFA expression (Fig. 2A) and tube formation were much more in the group treated with inflammatory factors for 14 days than that for 7 days (Fig. 2C and D). All these results suggested that inflammation promoted the pro-angiogenesis of PDLSCs.

### PDLSCs promoted angiogenesis through exosome-mediated pathway under inflammatory condition

**Figure 3:**
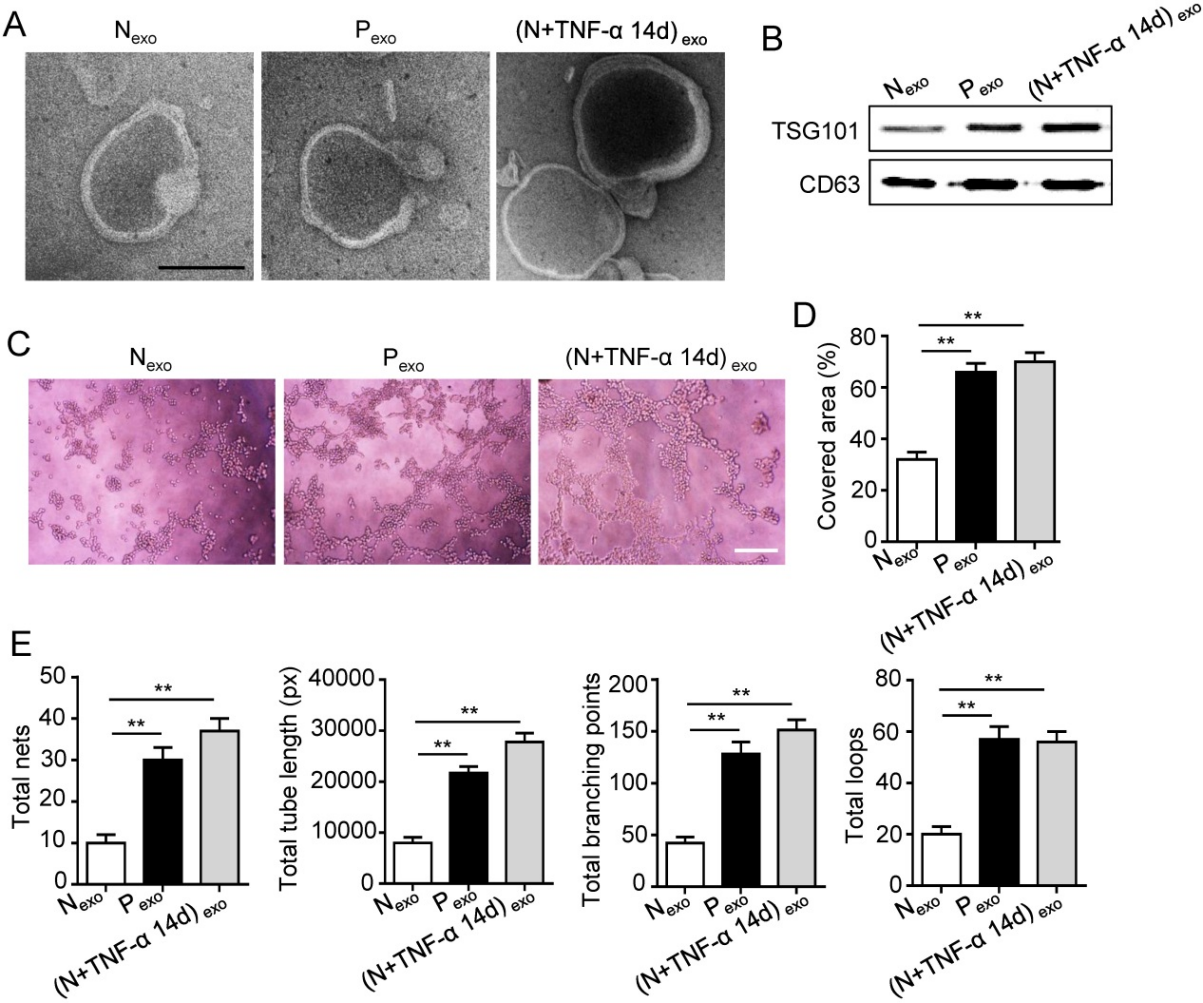
** Effect of PDLSCs on angiogenesis through exosome-mediated pathway under inflammatory condition.** (A) Exosomes secretion was detected using SEM. Scale bar = 100 nm. (B) Exosomes specific marker TSG101 and CD63 were examined by western blot. Tube formation of HUVECs (C) was observed under microscope after treatment with exosomes derived from PDLSCs, and total tube length, total branching points, total loops, covered area and total nets (D-E) were analyzed using Image J. Scale bar = 200 μm. **p<0.01. One-way analysis of variance (ANOVA).

**Figure 4:**
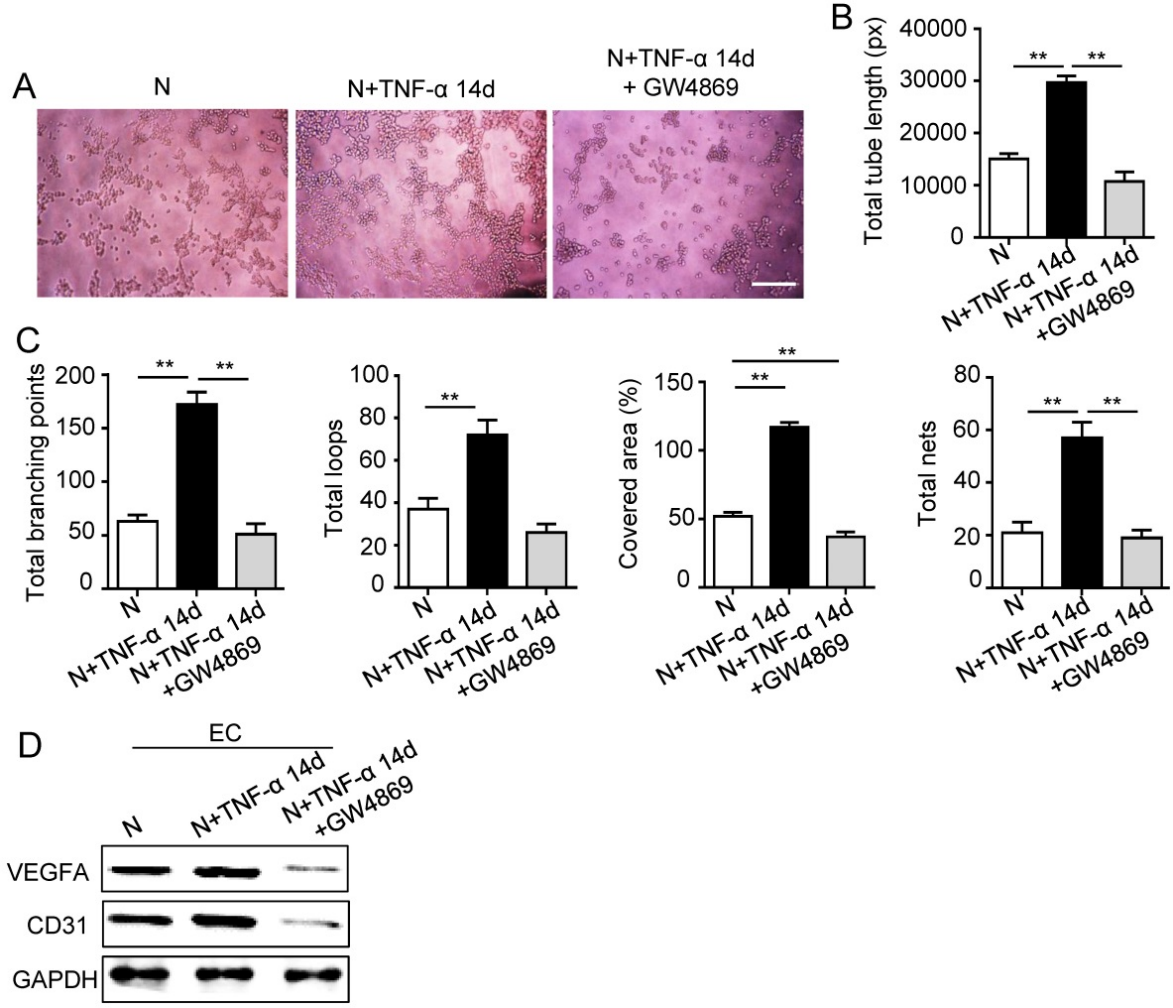
** Effect of PDLSCs on angiogenesis when exosome-mediated pathway was blocked under inflammatory condition.** Exosomes were blocked by GW4869. Tube formation of HUVECs (A) was observed under microscope, then total tube length, total branching points, total loops, covered area and total nets (B-C) were analyzed using Image J, and VEGF and CD31 of HUVECs (D) were detected by western blot after co-culturing with PDLSCs. Scale bar = 200 μm. **p<0.01. One-way analysis of variance (ANOVA).

The 14 days-treatment was used for further study because of its better effect on pro-angiogenesis compared to the 7 days-treatment. To explore whether inflammation influenced the exosomes secretion, PDLSCs were treated with inflammatory factors. Scanning electron microscope (SEM) showed that the quantity of exosomes was increased in inflammation group compared to control group (Fig. 3A). In addition, expressions of exosomes specific markers (TSG101, CD63) were higher in inflammation group than that in control group (Fig. 3B). The aforementioned data suggested that inflammation promoted exosomes secretion of PDLSCs. Furthermore, HUVECs treated with exosomes derived from inflamed PDLSCs exhibited better tube formation than control group (Fig. 3C-E). However, knocking- down exosomes of PDLSCs using GW4869 resulted in declined tube formation of HUVECs (Fig. 4A-C) and angiogenic markers (CD31, VEGFA) expression (Fig. 4D). Taken together, all these results indicated that PDLSCs promoted angiogenesis of HUVECs via exosome-mediated pathway under inflammatory micro-environment.

### Inflammation affected pro-angiogenesis of PDLSCs via regulating exosome-mediated transfer of VEGFA targeted by miR-17-5p

**Figure 5:**
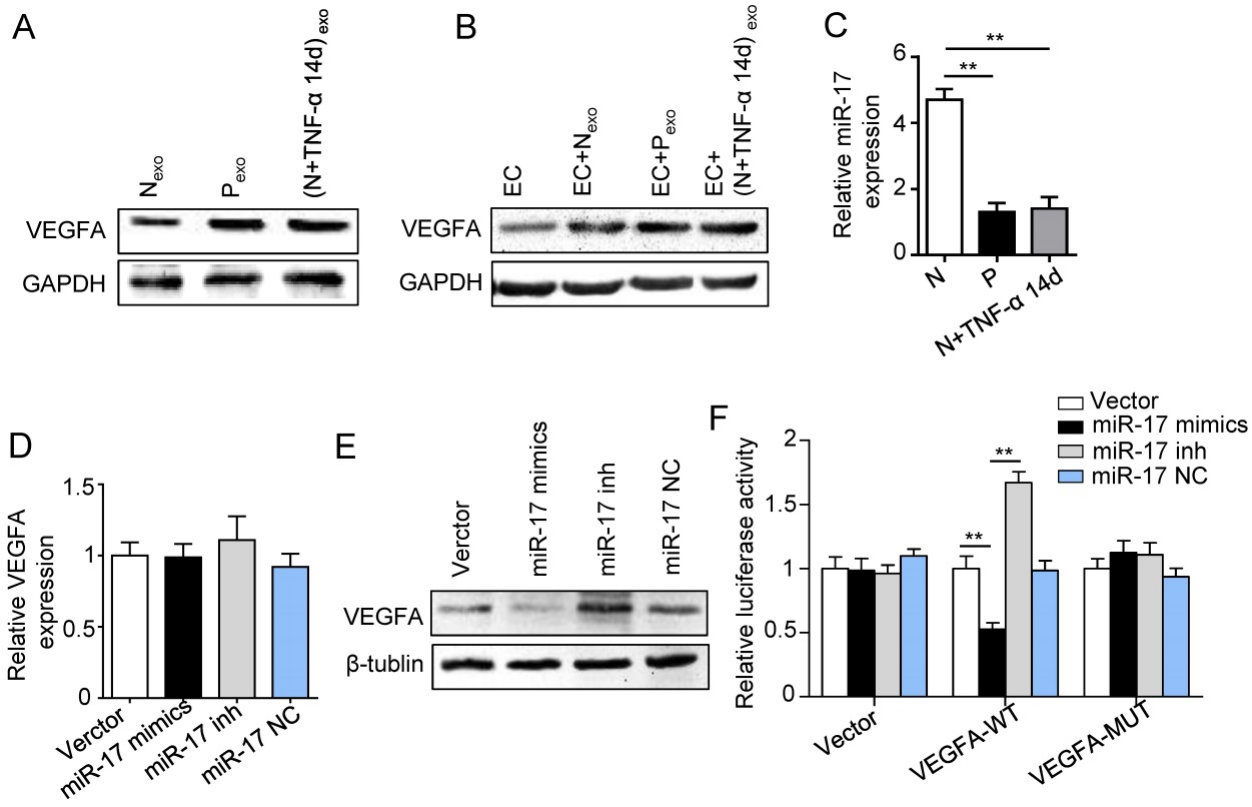
** Effect of inflammation on VEGFA transportation from PDLSCs to HUVECs, and verification of miR-17-5p targeting VEGFA.** (A) VEGFA expression in exosomes derived from was detected using western blot. (B) VEGFA expression in HUVECs was detected using western blot after treatment with exosomes derived from PDLSCs. (C) MiR-17-5p expression was detected using real-time PCR. VEGFA mRNA (D) and protein (E) were examined by real-time PCR and western blot respectively. (F) Luciferase activity of VEGF 3'-UTR was detected by luciferase report assay. **p<0.01. One-way analysis of variance (ANOVA).

In order to clarify whether exosomes transported VEGFA to HUVECs, proteins were analyzed by western blot. The VEGFA expression was much higher in exosomes derived from inflamed PDLSCs than control group (Fig. 5A). In addition, HUVECs also showed higher VEGFA expression when treated with exosomes derived from inflamed PDLSCs compared to normal PDLSCs (Fig. 5B), suggesting that PDLSCs transported VEGFA to HUVECs via exosomes secretion under inflammation.

**Figure 6:**
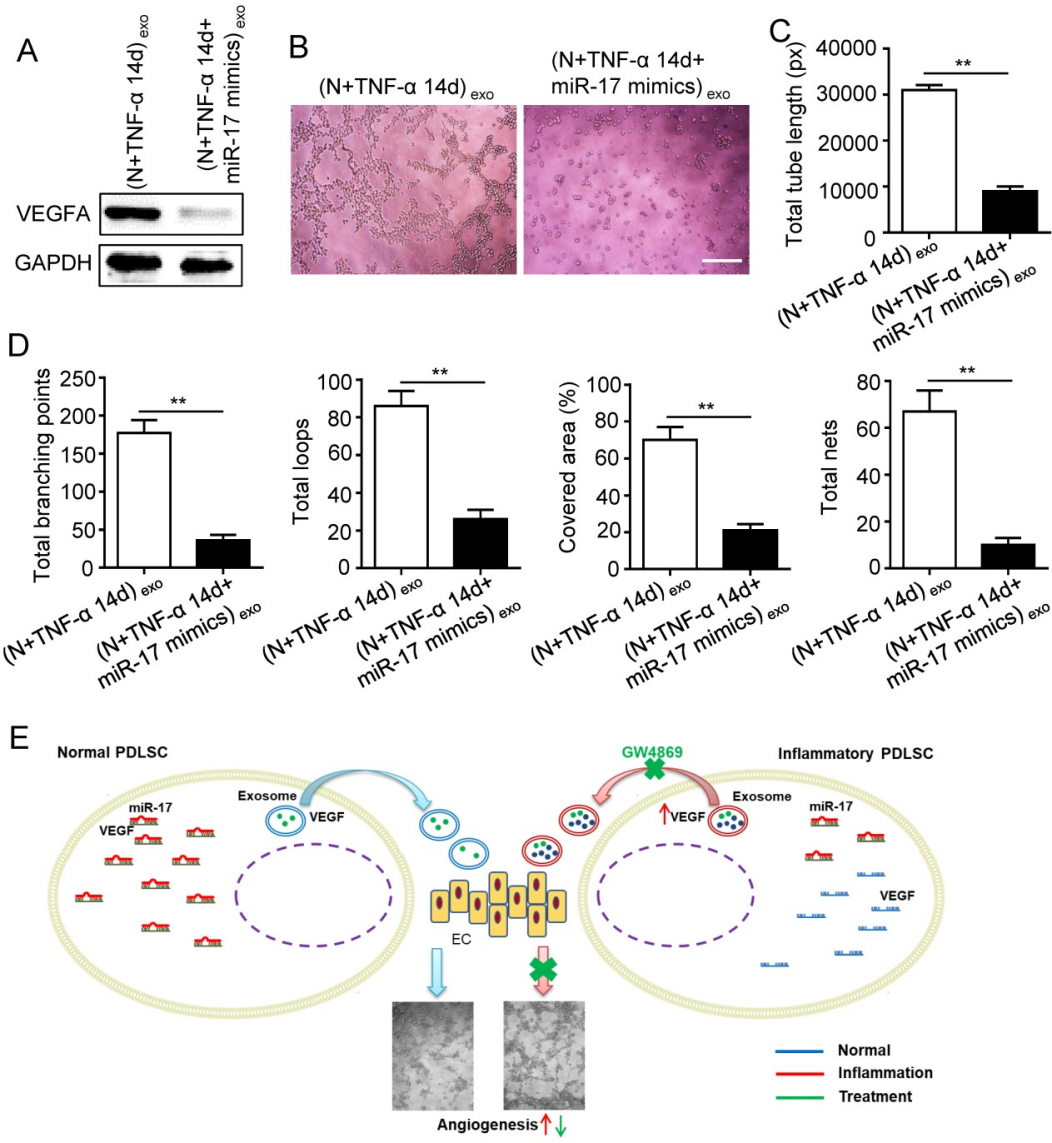
** Effect of inflammation on angiogenesis via regulating exosome-transferred VEGF signaling of PDLSCs targeted by miR-17-5p.** VEGFA expression (A) was detected in inflamed PDLSCs using western blot after transfecting miR-17 mimics, and tube formation (B) and total tube length, total branching points, total loops, covered area, total nets (C-D) were analyzed using Image J. (E) Schematic diagram of detailed mechanism. Scale bar = 200 μm. **p<0.01. Unpaired two-tailed Student's t-test.

Previous studies reported that miR-17 was related to angiogenesis and VEGF was a target of miR-17. To further identify the alteration of miR-17 and its effect on VEGF expression under inflammatory micro-environment, the gain and loss experiment was conducted through transfecting mimics and inhibitor of miR-17-5p into PDLSCs respectively (transfection efficiency see Fig. S2). The data showed that miR-17-5p was inhibited under inflammatory condition (Fig. 5C), but its alteration did not affect the secretion of exosomes (Fig. S3). Additionally, silence of miR-17-5p did not affect the expression of VEGFA mRNA (Fig. 5D) but significantly improved VEGFA protein expression (Fig. 5E), suggesting that miR-17-5p regulated VEGFA at post-transcriptional level. Luciferase reporter test showed that overexpression of miR-17-5p largely reduced the luciferase activity of VEGFA 3'-UTR, whereas knocking-down of miR-17 increased the reporter luciferase activity (Fig. 5F), confirming that VEGFA was a target of miR-17-5p. Furthermore, compared to control group, the VEGFA expression significantly decreased in exosomes derived from PDLSCs transfected by miR-17-5p mimics (Fig. 6A), meanwhile the tube formation of HUVECs was correspondingly declined (Fig. 6B-D). Taken together, all these data indicated that inflammation relieved the VEGFA suppression by inhibiting miR-17-5p of PDLSCs, and improved VEGFA transferring via exosomes secretion to facilitate angiogenesis of HUVECs (Fig. 6E).

## Discussion

Aberrant angiogenesis has been reported to be associated with the pathogenesis and progress of periodontitis 3, 5. Functions of ECs compromised under inflammatory microenvironment of periodontitis 25. Therefore, regulation of angiogenesis might be a candidate target for therapy of periodontitis. Gingiva and periodontal ligaments are neighbor tissues with close relationship, for example the main fibers of gingiva and periodontal ligaments cross over the alveolar ridge, the blood supply of periodontal ligaments partially derived from gingiva. Gingiva hyperemia is an obvious symptom of periodontitis, vascular alteration of gingiva has been investigated in the condition of periodontitis 5, 6. However, changes of vascularization of periodontal ligaments remain unknown. Therefore, it is significant to compare angiogenic conditions of these two tissues, and to determine the vascular alteration of periodontal ligaments to understand the mechanism of periodontitis and develop therapeutic approach. According to current study, periodontitis model was successfully established. Furthermore, similar to gingiva, vascularization of periodontal ligaments was also enhanced in periodontitis, suggesting that periodontal ligaments shared similar angiogenic disorder with gingiva under inflammation. However, the mechanism of vascular alteration remains unknown.

PDLSCs are a type of dental stem cells residing in periodontal ligaments, which participate in regulation of periodontal tissues regeneration 26. Apart from feature of osteogenic capacity, PDLSCs has been investigated to modulate angiogenesis of ECs 4, 9. An indirect communication, such as paracrine, might be the crucial pathway involved in the mechanism, because PDLSCs and ECs could not directly interact with each other physiologically. Therefore, a transwell indirect co-culture system was employed to mimic cell-to-cell communication. Functions of PDLSCs are affected by inflammatory environment resulted from periodontitis, such as proliferative capacity, osteogenic potential and regulatory ability 27-29. In our study, VEGFA, the vascular specific marker, was up-regulated in PDLSCs derived from periodontitis, suggesting that inflammation might affect the pro-angiogenesis of PDLSCs. Furthermore, we found that the pro-angiogenic ability of PDLSCs was improved under inflammatory condition with a time-dependent manner, which corresponded to the results of gradually enhanced vascularization in periodontitis 3, 5. Thus, we confirmed that inflammatory micro-environment in periodontitis might be the key regulator on pro-angiogenesis of PDLSCs, and inflamed PDLSCs regulated HUVECs via paracrine dependent pathway.

Exosomes is a well-known paracrine mediator to execute mission of intercellular contact 13 and is associated with the change of micro-environment 30. It has been reported that exosomes secretion and contents were under control of inflammatory microenvironment 31, 32. We found that the secretion of exosomes was increased under inflammatory condition, indicating that the exosomes secretion was altered in PDLSCs of periodontitis. Furthermore, treatment with inflammatory exosomes promoted the angiogenesis of HUVECs, whereas blocking exosomes secretion abolished the pro-angiogenesis of inflamed PDLSCs. Therefore, we confirmed that inflammation promoted pro-angiogenesis of PDLSCs via exosomes mediated pathway.

Exosomes is a carrier to transport active factors intercellularly, such as functional protein and small RNA^13^. In current study, expression of VEGFA was increased in exosomes derived from inflamed PDLSCs, suggesting the important role of exosome-VEGFA in angionenic regulation. However, the up-stream signaling regulating VEGFA expression under inflammation remains elusive. MicroRNAs have been widely investigated to regulate vascularization through various signaling 33, 34. MiR-17, a potential inhibitor of VEGF, has been reported to be associated with periodontal regeneration 21, 22, 24 and tissue angiogenesis 23. Nevertheless, the role of miR-17 on pro-angiogenesis of PDSLCs in periodontitis remains unknown. We found that miR-17-5p was decreased under inflammation with a time-dependent manner, indicating a significant role of miR-17 in periodontitis. Additionally, the fact of miR-17-5p targeting on VEGFA was verified. Moreover, overexpression of miR-17-5p in inflamed PDLSCs resulted in decreased exosome-VEGFA and declined tube formation. Meanwhile, the effect of miR-17-5p on exosomes secretion was eliminated, confirming that the reduction of exosome-VEGFA credited to VEGFA suppression but not exosomes inhibition triggered by miR-17-5p. In addition, in our study, exosomes secretion and miR-17-5p/VEGFA signaling were both affected by inflammatory micro-environment, suggesting that up-regulation of exosomes secretion and VEGFA expression might synergistically contribute to increased vascularization of periodontal ligaments in periodontitis.

All the aforementioned data suggested that vascularization of periodontal ligaments was up-regulated in periodontitis, which might credit to the fact that PDLSCs regulated angiogenesis of HUVECs via exosome-mediated transfer of VEGFA targeted by miR-17-5p.

## Supplementary Material

Supplementary figures.

## Abbreviations

PDLSCs: periodontal ligament stem cells
VEGF: vascular endothelial growth factor
HUVECs: human umbilical vein endothelial cells
MSCs: Mesenchymal stem cells
miRNAs: micro-RNAs
