# PDLSCs Regulate Angiogenesis of Periodontal Ligaments via VEGF Transferred by Exosomes in Periodontitis: Erratum

**DOI:** 10.7150/ijms.74583

**Published:** 2022-05-03

**Authors:** Zhang Zhang, Yi Shuai, Feng Zhou, Jikai Yin, Jiachen Hu, Songlin Guo, Yan Wang, Wenjia Liu

**Affiliations:** 1 Department of General Surgery, Tang Du Hospital, Fourth Military Medical University, Xi'an, Shaanxi 710032, People's Republic of China;; 2 Department of Stomatology, Jinling Hospital, Medical School of Nanjing University, Nanjing, Jiangsu 210002, People's Republic of China;; 3 State Key Laboratory of Military Stomatology & National Clinical Research Center for Oral Diseases & Shaanxi International Joint Research Center for Oral Diseases, Center for Tissue Engineering, School of Stomatology, Fourth Military Medical University, Xi'an, Shaanxi 710032, People's Republic of China;; 4 Department of Stomatology, General Hospital of Eastern Theater Command, PLA, Nanjing, Jiangsu 210002, People's Republic of China;; 5 Xi'an Institute of Tissue Engineering and Regenerative Medicine, Xi'an, Shaanxi 710032, People's Republic of China;; 6 Department of Clinical Laboratory, The First Affiliated Hospital of Xi'an Medical University, Xi'an, Shaanxi 710032, People's Republic of China.

The authors regret that the original version of our paper unfortunately contained some incorrect representative images. The transwell images of tube formation in Figure 3C had been misused during figure assembly. The correct version of the Figure 3C appears below.

The authors confirm that the corrections made in this erratum do not affect the original conclusions. The authors apologize for any inconvenience that the errors may have caused.

## Figures and Tables

**Figure 3:**
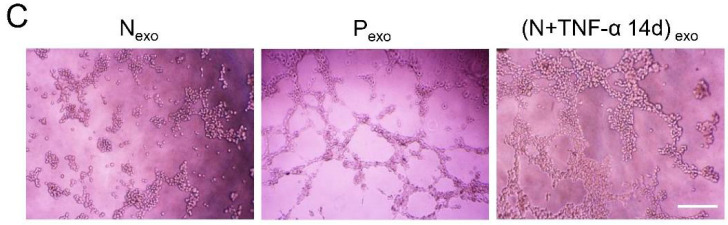
(C) Tube formation of HUVECs was observed under microscope after treatment with exosomes derived from PDLSCs. Scale bar = 200 μm.

