# Supplementary material for: PDLSCs Regulate Angiogenesis of Periodontal Ligaments via VEGF Transferred by Exosomes in Periodontitis
Source: Int J Med Sci. 2020 Feb 10;17(5):558–67. doi: 10.7150/ijms.40918 (PMC7085218; doi:10.7150/ijms.40918)
Supplement: Supplementary file 1 — Supplementary figures. [file ijmsv17p0558s1.pdf]

PDLSCs regulate angiogenesis of periodontal ligaments via VEGF transferred by exosomes in periodontitis

Zhang Zhang[1,†,*], Yi Shuai[2,3,†], Feng Zhou[4,5,†], Jikai Yin[1], Jiachen Hu[4,5], Songlin Guo[1], Yan Wang[6], Wenjia Liu[4,5,*]

[1.] Department of General Surgery, Tang Du Hospital, Fourth Military Medical University, Xi’an, Shaanxi 710032, People’s Republic of China;

[2.] Department of Stomatology, Jinling Hospital, Medical School of Nanjing University, Nanjing, Jiangsu 210002, People’s Republic of China;

[3.] Department of Stomatology, General Hospital of Eastern Theater Command, PLA, Nanjing, Jiangsu 210002, People’s Republic of China;

[4.] State Key Laboratory of Military Stomatology＆National Clinical Research Center for Oral Diseases＆Shaanxi International Joint Research Center for Oral Diseases, Center for Tissue Engineering, School of Stomatology, Fourth Military Medical University, Xi’an, Shaanxi 710032, People’s Republic of China;

[5.] Xi'an Institute of Tissue Engineering and Regenerative Medicine, Xi’an, Shaanxi 710032, People’s Republic of China;

[6.] Department of clinical laboratory, The first affiliated hospital of Xi’an Medical University, Xi’an, Shaanxi 710032, People’s Republic of China;

Figure S1

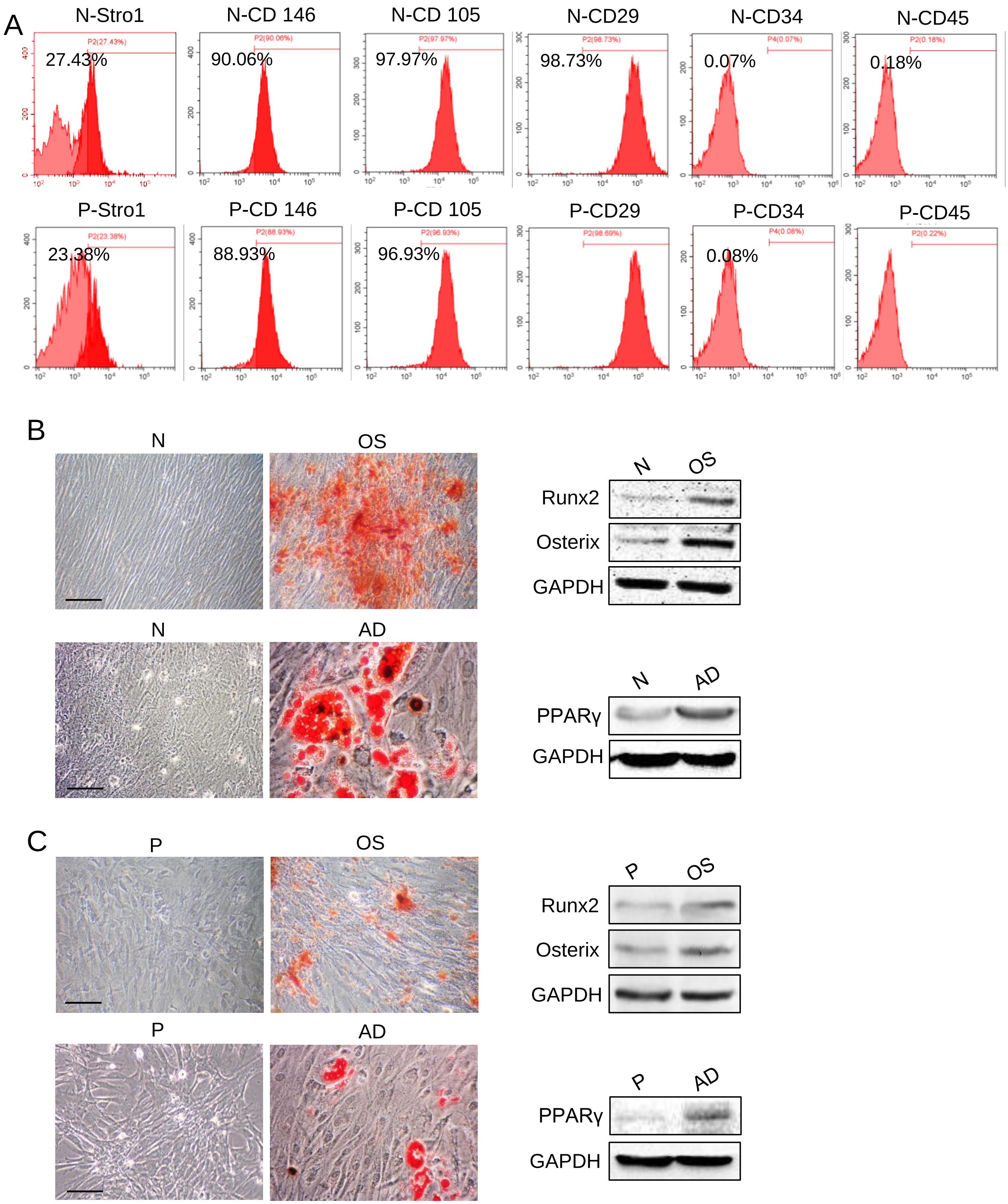

A
N-Stro1
27.43%
N-CD 146
90.06%
N-CD 105
97.97%
N-CD29
98.73%
N-CD34
0.07%
N-CD45
0.18%
P-Stro1
23.38%
P-CD 146
88.93%
P-CD 105
96.93%
P-CD29
P-CD34
0.08%
P-CD45
B
N
OS
N
OS
Runx2
Osterix
GAPDH
N
AD
N
AD
PPARγ
GAPDH
C
P
OS
P
OS
Runx2
Osterix
GAPDH
P
AD
P
AD
PPARγ
GAPDH

Fig.S1. Identification of PDLSCs. (A) Surface markers of PDLSCs from normal and periodontitis were analyzed using flow cytometry. Mineralized nodules, lipid droplets formation, osteogenic and adipogenic makers of PDLSCs from normal (B) and periodontitis (C) were determined by Alizarin Red staining, Oil Red O staining and western blot respectively. Scale bar = 50 μm.

Figure S2

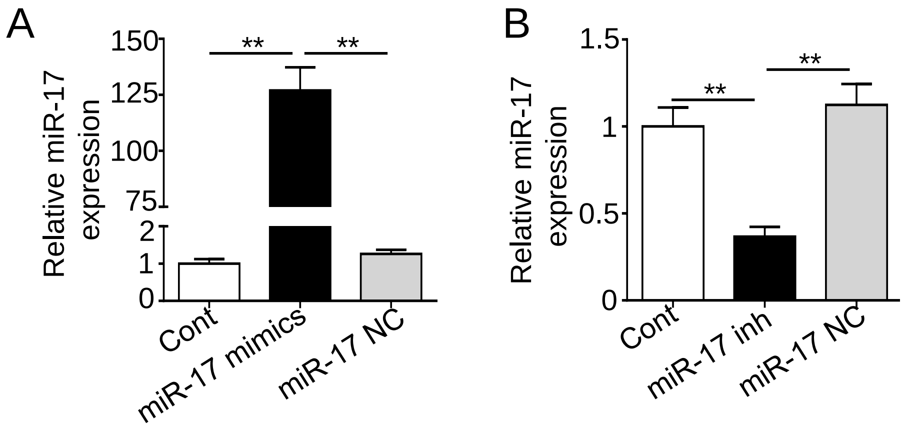

Fig.S2. The efficiency of miR-17 transfection. Efficiency of miR-17-5p mimics (A) and inhibitor (B) transfection were detected using real-time PCR. **$p<0.01$. One-way analysis of variance (ANOVA).

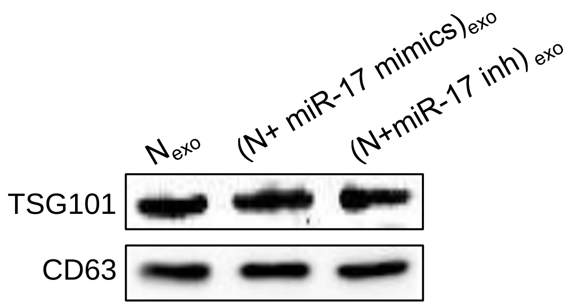

Fig.S3. Effect of miR-17-5p on exosome secretion. Exosome specific markers TSG101 and CD63 were detected after PDLSCs being transfected with miR-17-5p mimics (A) and inhibitor (B) using western blot.
